# Attitudes of nursing students toward older adults: a systematic review and meta-analysis

**DOI:** 10.3389/fpubh.2026.1813734

**Published:** 2026-06-04

**Authors:** Wei Zhang, Xueke Hui, Lili Wei, Yunhua Wang, Zhirui Li, Xihao Zhang, Mengting Li

**Affiliations:** 1 School of Health Management, Gansu University of Chinese Medicine, Lanzhou, China; 2 Lanzhou Maternal and Child Health Care Hospital, Lanzhou, China; 3 School of Management, Lanzhou University, Lanzhou, China

**Keywords:** attitude, geriatric care, meta-analysis, nursing students, systematic review

## Abstract

**Background:**

Global population aging has precipitated an increased demand for professional geriatric care while facing a shortage of nursing personnel and inconsistent attitudes toward older adults care among nursing students. A systematic review and meta-analysis were conducted to examine nursing student’s attitudes toward older adults care and the factors that influence these attitudes in order to optimize the education of geriatric nurses.

**Methods:**

This meta-analysis was reported following the PRISMA 2020 Checklist. PubMed, Web of Science, Embase, Cochrane Library, China National Knowledge Infrastructure, Wanfang data, VIP, and Chinese Biomedical Literature Service System were systematically searched from inception to July 4, 2024, to identify cross-sectional surveys reporting nursing student’s attitudes toward older adults. Meta-analysis was performed using Stata 17.0 software after two researchers independently screened the literature, extracted information, and evaluated the risk of bias in the included studies. Subgroup and sensitivity analyses were performed to address heterogeneity. Funnel plots and Egger’s test were used to assess the publication bias.

**Results:**

Sixty-four studies of nursing student’s attitudes toward geriatric care from 11 countries, 47 using KAOP 34–238 and 17 using KAOP 34–204, involved 19,933 and 4,399 nursing students, respectively. Fifty-six studies were assessed as being of high or moderate quality, while eight studies were classified as low quality. Regarding nursing students’ attitudes toward older adults, the results of the meta-analysis showed that the KAOP 34–238 score was 146.41 (95% CI: 141.16–151.66, *I*^2^ = 99.8%), and the KAOP 34–204 score was 130.95 (95% CI: 125.53–136.39, *I*^2^ = 99.5%). Subgroup analyses of these predictor variables revealed that nursing students showed statistically significant differences in the following factors (*p* < 0.05): gender, having lived with and caring for an older person, having taken a course in geriatric care, selecting nursing as their first choice, and student grade level.

**Conclusion:**

The attitude of nursing students toward older adults is generally positive. Future studies should be designed with higher quality to explore the relationship between attitudes toward older people and nursing students’ willingness to work in geriatric care.

**Systematic review registration:**

CRD42022348244.

## 1 Introduction

Population aging is one of the most defining global trends of our era (1). In 2024, individuals aged 60 and above accounted for 14.5% of the global population, marking an increase of 2.3 percentage points since 2015 (2). The United Nations predicts that the world population will peak at around 10.3 surpassing the population of those aged 18 and under (3). China, in particular, has entered a phase of deep aging, with the proportion of older individuals expected to surpass 20% by 2030, thus transitioning into a super-aging society. By 2060, this proportion is projected to reach approximately 37.4% (4). Similarly, in the European Union (EU-27), the median age is expected to increase to 48.2 years between 2019 and 2050, with nearly half a million centenarians expected by mid-century (5).

Aging is associated with a wide range of complex health challenges, including reduced mobility, loss of appetite, chronic pain, dementia, cancer, and mental health disorders (6, 7). In the United Kingdom, by 2024, approximately 32% of individuals aged 65 and above cannot care for themselves.

In China, the number of older adults with disabilities is rising at an accelerated pace. The disabled or semi-disabled older population in both urban and rural areas is expected to increase from 45.636 million in 2020 to 69.526 million in 2030 and further to 126.06 million in 2050 (8, 9). Additionally, the global prevalence of dementia currently exceeds 55 million cases, with projections indicating that dementia prevalence in Europe will double by 2050 (10, 11). Furthermore, older adults often experience multi-morbidity, with hypertension, diabetes, and depression being among the most frequently observed comorbid conditions (12, 13).

The rapid aging of the global population has created a significant and urgent need for professional healthcare services. In China, there are more than 500,000 nurses, yet this workforce remains insufficient to meet the care needs of 260 million older individuals and over 40 million disabled and semi-disabled older adults (14). Similarly, the 2020 State of World Nursing report estimated a global shortage of 5.9 million nurses, with the deficit primarily concentrated in low- and middle-income countries (15). Alarmingly, this shortfall accounts for over 50% of the total global healthcare workforce gap. The World Health Organization (WHO) projects that an additional 9 million nurses will be needed by 2030 to meet sustainable healthcare and well-being goals (15).

Attitudes are defined as acquired, relatively stable tendencies that shape evaluative responses, ideas,and behaviors. Various factors—including cultural background, age, gender, educational level, field of study, professional affinity for nursing, and past interactions with older adults—collectively influence nursing students’ attitudes toward older adults (16–18). It is important to distinguish between attitudes toward older adults (the focus of this review) and intentions to work in geriatric care; while related, these are distinct constructs. Studies across many countries have consistently shown that geriatric care remains an unpopular career choice among nursing students. In Sri Lanka and Turkey, nursing students’ willingness to work with older adults remains low (19, 20). In dementia care facilities, the manifestation of challenging behaviors—such as wandering, agitation, and persistent attempts to leave the premises—strains caregivers’ coping capacity and increases the complexity of care provision. Furthermore, a cross-sectional survey involving 1,796 nursing students across six European countries revealed a low level of interest in care for older adults, with an average score of 20.5 on a 0–100 scale (21). Additionally, some nursing students perceived caring for older people as burdensome, monotonous, and unproductive while also reporting difficulties in communication (22, 23). However, some studies present an alternative view. A systematic review of 377 undergraduate nursing students found that students had generally positive attitudes toward older adults, with a mean Kogan score of 131.04, exceeding the benchmark of 102. These results suggest that being female, having clinical placements in hospitals and care homes, and having previous contact with older individuals were all associated with more positive attitudes (24). Similarly, a cross-sectional study of 1,179 Austrian nurses indicated that their attitudes toward people aged 80 and over ranged from neutral to positive, particularly regarding geriatric care (25).

Likewise, a study conducted in a tertiary care facility in Ghana examined hospitalized older patients and found that, while nurses exhibited positive attitudes, there was no significant correlation between educational level and attitude positivity (26).

This study conducted a comprehensive systematic review and meta-analysis of nursing students’ attitudes toward older adults, utilizing the KAOP scale, a widely recognized instrument for assessing attitudes toward aging. The findings aim to provide an empirical foundation for refining nursing education and training, ensuring an optimal balance between workforce supply and demand in geriatric care, and facilitating the progressive development of nursing services.

## 2 Materials and methods

### 2.1 Registration

This study followed the Preferred Reporting Items for Systematic Reviews and Meta-Analyses (PRISMA) guidelines. The protocol was registered in the Prospective Register for Systematic Reviews (PROSPERO) (CRD42022348244). Supplementary Table 1 provides the PRISMA 2020 checklist items.

### 2.2 Search strategy

We systematically searched eight English and Chinese databases, including PubMed, Web of Science, Embase, Cochrane Library, China National Knowledge Infrastructure (CNKI), Wanfang data, VIP, and the Chinese biomedical literature service system from inception to July 4, 2024. The Medical Subject Headings (MeSH) terms and keywords used were: “Students, Nursing,” Geriatric Nursing,” and “Attitude.” Supplementary Appendix 1 details the complete search strategy.

Additionally, we screened the reference lists of the included articles and the relevant systematic reviews and meta-analyses to identify additional studies that meet the inclusion criteria. Due to the authors’ linguistic capabilities, only studies published in English or Chinese were included.

### 2.3 Inclusion and exclusion criteria

For nursing students’ attitudes toward older adults, the inclusion criteria for this study were as follows: (1) cross-sectional studies; (2) respondents were undergraduate nursing students in any form of post-secondary program (including undergraduate, junior college, and vocational who had not yet graduated at the time of the survey, with no restrictions on school type or year of study; (3) the instrument used to measure nursing students’ attitudes toward older adults was Kogan’s Attitudes toward Old People (KAOP) scale, with scores ranging from 34 to 238 or 34–204; and (4) the total score of nursing students’ attitudes toward older adults was explicitly reported.

The exclusion criteria included: (1) duplicate published studies; (2) publications in languages other than English or Chinese; (3) studies with missing relevant data; (4) studies based on the same population, in which case only the most recent study was included.

### 2.4 Study selection and data extraction

Two reviewers independently screened the titles and abstracts of the studies according to the inclusion and exclusion criteria to identify eligible full-text articles. Data was extracted from the included studies using a standardized form that collected essential information, such as first author, year of publication, study region, survey year, sampling method, sample size, gender distribution, academic grade, source of participants, and total KAOP score.

The KAOP scale consists of 34 items, including 17 statements reflecting positive and 17 reflecting negative attitudes. These items were categorized into two dimensions: KAOP+ (appreciation) and KAOP- (prejudice). The scale employs a seven-point Likert response format, yielding total scores ranging from 34 to 238. A median score of 136 serves as the threshold, with scores higher than 136 indicating a positive attitude toward older adults and below 136 indicating a negative attitude.

However, some studies adopted a six-point Likert scale with response options of “strongly disagree,” “disagree,” “slightly disagree,” “slightly agree,” “agree,” and “strongly agree,” scored from 1 to 6, respectively. In these cases, the total KAOP scores ranged from 34 to 204, with a median threshold of 102 (27). Similar to the seven-point scale, scores above 102 indicated a positive attitude, while those below 102 suggested a more negative attitude toward older adults. After data extraction, the two reviewers cross-checked their findings. Any discrepancies were resolved through discussion, and if necessary, a third reviewer was consulted to reach a consensus.

### 2.5 Methodological quality assessment

The quality of the included studies was evaluated using the tool recommended by the Agency for Healthcare Research and Quality (AHRQ) (28). Since item 11 of the tool pertains to potential bias due to lack of follow-up and applies only to prospective studies, it was excluded from this analysis.

The remaining 10 items, presented in Table 1, were assessed. Each item was scored as either “yes” (1 point) or “no/'unclear” (0 points). The overall study quality was graded as low (0—3 points), 145moderate (4–7 points) or high (8–10 points). Two reviewers independently assessed the risk of bias in the included studies and cross-checked the results.

**Table 1:** List of AHRQ items.

Number	Item
1	Define the source of information (survey, record review)
2	List inclusion and exclusion criteria for exposed and unexposed subjects (cases and controls) or refer to previous publications.
3	Indicate the time period used for identifying patients.
4	Indicate whether or not subjects were consecutive if not population-based
5	Indicate if evaluators of subjective components of the study were masked to other aspects of participants, status.
6	Describe any assessments undertaken for quality assurance purposes (e.g., test/retest of primary outcome measurements)
7	Explain any patient exclusions from the analysis.
8	Describe how confounding was assessed and/or controlled;
9	If applicable, explain how missing data were handled in the analysis.
10	Summarize patient response rates and completeness of data collection.

### 2.6 Data analysis and synthesis

In this meta-analysis, the statistical effect size for nursing students, attitudes toward older adults was represented by the total score of the attitude measures. Statistical analysis was performed using Stata 17.0 software. Heterogeneity among the included studies was assessed using the chi-square test (*α* = 0.1), and the degree of heterogeneity was quantified using the *I*^2^ statistic. If *I*^2^ < 50% and *p* > 0.10, the studies were considered to have no significant heterogeneity and a fixed-effects model was applied for the meta-analysis. Conversely, if significant heterogeneity was present, a random-effects model was used.

To investigate nursing students’ attitudes toward older adults, this study identified nine predictor variables for subgroup analyses: four were pre-specified in the PROSPERO protocol [gender (29–33), place of origin (29, 32, 33), academic year (34–36), and only-child status (29, 32, 34–36)] and five were examined post-hoc [experience in caring for older adults (37–40), living with older adults (29, 32, 33, 41), completion of geriatric nursing course (33, 38, 41, 42), choosing nursing as first-choice career (33, 43–46), and geographical location (29, 47–49)].

Thus, the subgroup analyses included nine predictor variables: gender (male vs. female), student origin (urban vs. rural), one-child status (yes vs. no), experience of living with older adults (yes vs. no), experience of caring for older adults (yes vs. no), completion of a geriatric training courses (yes vs. no), whether they have made caring for older adults their first choice (yes vs. no), academic year (first, second, third or fourth year), and country (China vs. other countries). To determine whether differences between subgroups were statistically significant, meta-regression was conducted with a *p*-value for interaction < 0.05 considered significant.

Sensitivity analyses were conducted to using leave-one-out analysis assess the robustness of the results. When 10 or more analyses were included in an outcome, publication bias was assessed using Egger’s linear regression test (50) to assess publication bias and funnel plots were generated. A *p*-value of < 0.05 was considered statistically significant.

## 3 Results

### 3.1 Search results

**Figure 1:**
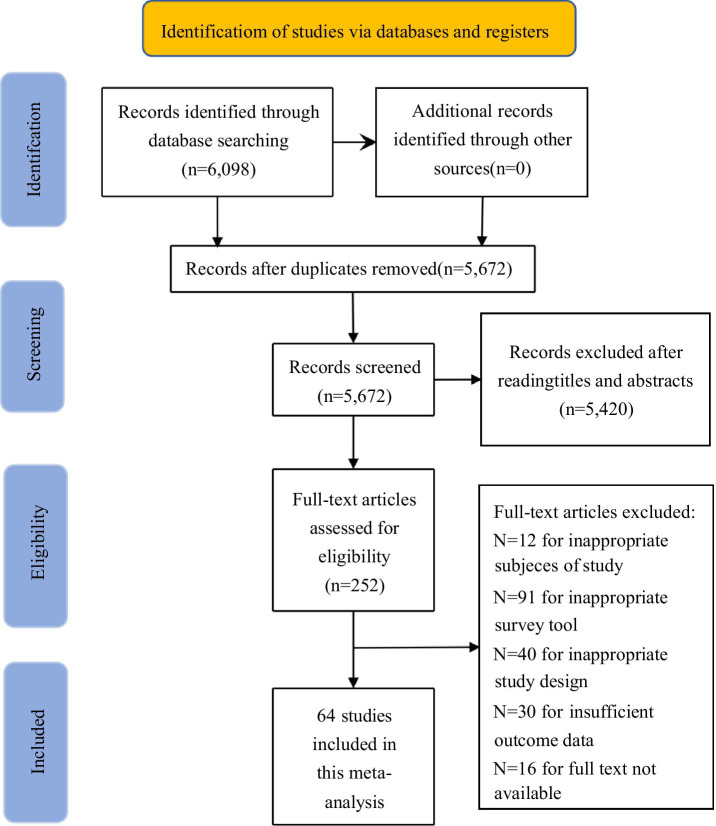
PRISMA flow diagram of literature search and selection.

The initial search yielded 6,098 studies. After removing duplicates, the titles and abstracts of 5,672 studies were screened. We reviewed 252 full-text manuscripts, and 64 studies were ultimately included. Of these, 47 used the KAOP scale with a total score range of 34–238, while 17 used the KAOP scale with a range of 34–204. The study selection flow chart is illustrated in Figure 1.

### 3.2 Study characteristics

Details of the included studies are summarized in Table 2. A total of 47 studies utilized the KAOP scale with a total score range of 34–238 (19, 29, 38, 41, 46, 51–82), while 17 studies (47, 48, 83–90) used the KAOP scale with a total score range of 34–204. Among studies using the 34–238 KAOP scale, 19,933 nursing students were surveyed, with sample sizes ranging from 51 to 1,335. Most studies (93.6%, *n* = 44) were conducted in China (29, 38, 41, 46, 51–54, 56–62, 64–82), while the remaining 6.4% (*n* = 3) (19, 55, 63) were from Sri Lanka, Thailand and Malaysia. Surveys were conducted between 2009 and 2022 using various sampling methods, including convenience sampling, stratified random sampling, stratified cluster sampling, cluster sampling, and purposive sampling. Additionally, 44 studies reported KAOP scores separately for male and female nursing students.

**Table 2:** Summary characteristics of included studies about nursing students’ attitudes towards older people.

						Gender		
Author, Year	Area	Time of survey	Sampling method	Sample size (n)	Level of students	Male [*n* (%)]	Female [*n* (%)]	Age
KAOP34-238 Guo et al. (27)	Nantong, China	2017.5–7	Convenience sampling	853	Undergraduate students	39 (4.57%)	814 (95.43%)	21.41 ± 1
Rathnayake et al. (19)	Sri Lanka	2015.4	/	98	Undergraduate students	36 (36.73%)	62 (63.27%)	24.5 ± 1.
Runkawatt et al. (64)	Thailand	2009	Convenience sampling	241	/	/	/	20.61 ± 1
Elias et al. (56)	Malaysia	2018.3–4	Convenience sampling	291	Undergraduate bachelor’s degree students	47 (16.15%)	244 (83.85%)	21.68 ± 1
Zhang et al. (81)	Tianjin, China	2015.11–12	Cluster sampling	382	Undergraduate students	32 (8.38%)	350 (91.62%)	20.4
Chen et al. (53)	Zhengzhou, China	2018.5–7	Convenience sampling	317	Postgraduate students, Undergraduate students, Junior college students	45 (14.20%)	272 (85.80%)	21.28 ± 2
Chen et al. (52)	Zhejiang, China	2012.4–5	Stratified random cluster sampling	997	Junior college students	27 (2.71%)	970 (97.29%)	20.00 ± 0
Chen et al. (54)	China	2016.12	Stratified random sampling	443	Junior college students	17 (3.84%)	426 (96.16%)	17.59 ± 1
Chen et al. (55)	Sichuan, China	/	Cluster sampling	224	Undergraduate students	20 (8.93%)	204 (91.07%)	20.99 ± 0
Cui et al. (42)	China	2019.1	Stratified cluster sampling	480	Junior college students	117 (24.38%)	363 (75 62%)	/
Guo et al. (57)	China	2016.10	Convenience sampling	233	Undergraduate students	/	/	/
Ji et al. (58)	China	/	Convenience sampling	380	Junior college students	26 (6.84%)	354 (93.16%)	20.04 ± 1
Li et al. (59)	Zhaoqing, China	/	Convenience sampling	641	Junior college students	35 (5.46%)	606 (94.54%)	/
Li (60)	Changchun, China	/	Stratified sampling	774	Undergraduate students, Junior college students	52 (6.72%)	722 (93.28%)	19.36 ± 1
Liu et al. (62)	Zhejiang, China	/	Random sampling	374	Undergraduate students	24 (6.42%)	350 (93.58%)	/
Liu (61)	China	/	/	340	Undergraduate students, Junior college students	36 (10.59%)	304 (89.41%)	/
Liu (39)	Sichuan, China	/	Stratified cluster sampling	1,158	Undergraduate students, Junior college students	70 (6.04%)	1,088 (93.96%)	/
Luo et al. (50)	China	2014.5	Random sampling	231	Junior college students	13 (5.63%)	218 (96.37%)	20.06
Qi et al. (63)	Shandong, China	2020.4	Convenience sampling	182	Undergraduate students	24 (13.19%)	158 (86.81%)	20.28 ± 0
Sun et al. (65)	Tianjin, China	/	Convenience sampling	348	Undergraduate students	42 (12.07%)	306 (87.93%)	20.01 ± 1
Sun (66)	Changchun, China	/	Convenience sampling	136	Undergraduate students	13 (9.56%)	123 (90.44%)	20.58 ± 0
Sun et al. (67)	Guangzhou, China	2015.3–4	Convenience sampling, Cluster sampling	180	Undergraduate students	24 (13.33%)	156 (86.67%)	21.66 ± 0
Tian et al. (68)	Changsha, China	/	Convenience sampling	185	Junior college students	0	185 (100.00%)	17.87 ± 0
Tian et al. (69)*	Changsha, China	/	Convenience sampling	190	Junior college students	0	190 (100.00%)	17.88 ± 0
Wan et al. (70)	Sichuan, China	/	Cluster sampling	51	Postgraduate students	3 (5.88%)	48 (94.12%)	31 ± 9
Wang et al. (71)	Fuzhou, China	2016.9–2017	Stratified random sampling	563	Undergraduate students, Junior college students	9 (1.60%)	554 (98.40%)	19.09 ± 2
Wei et al. (72)	Wuhan, China	/	Cluster sampling	371	Undergraduate students	0	371 (100.00%)	22.34 ± 1
Wu et al. (73)	China	2015.6–7	Convenience sampling	240	Undergraduate students	32 (13.33%)	208 (86.67%)	19.74 ± 0
Xu et al. (74)	Jiangsu, Taizhou, China	/	Cluster sampling	674	Junior college students	73 (10.83%)	601 (89.17%)	20.13 ± 0
Yan et al. (76)	Sichuan, Chengdu, China	/	Convenience sampling	551	Undergraduate students	67 (12.16%)	484 (87.84%)	/
Yan et al. (75)	Sichuan, Chengdu, China	/	Convenience sampling	542	Undergraduate students	66 (12.18%)	476 (87.82%)	/
Yang et al. (77)	Tianjin, China	2015.11–12	Convenience sampling	382	Undergraduate students	32 (8.38%)	350 (91.62%)	/
Yang et al. (78)	Hubei, Wuhan, China	/	Convenience sampling	258	Undergraduate students	/	/	/
Yao et al. (79)	Shanxi, China	/	Stratified random cluster sampling	373	Undergraduate students	29 (7.77%)	344 (92.23%)	/
Zhang et al. (80)	Anhui, Tonglin, China	2019.3–12	Stratified random sampling	312	Junior college students	16 (5.13%)	296 (94.87%)	19.53 ± 1
Zhou (82)	Xi’an, China	/	Random sampling	202	Undergraduate students	12 (5.94%)	190 (94.06%)	/
Zhu et al. (83)	Zhejiang, Hangzhou, China	/	Convenience sampling	480	Undergraduate students	14 (2.92%)	466 (97.08%)	22
Tao et al. (97)	Beijing, China	2022.4	Convenience sampling	582	Secondary school students, junior college students	163 (28.00%)	419 (71.99%)	/
Feng et al. (98)	Sichuan, Chengdu, China	/	Purpose sampling, convenience sampling	200	junior college students	13(6.50%)	187 (93.50%)	19.20 ± 2
Huang et al. (99)	Zhejiang, Ningbo, China	2012.4–5	Stratified random sampling	997	junior college students	27(2.71%)	970 (97.29%)	20 ± 0.9
Liu et al. (100)	Henan, Xuchang, Chi na	/	convenience sampling	108	junior college students	0(0.00%)	100 (100%)	17.58 ± 1
Li et al. (59)	Guangdong, Zhaoqing, Ch ina	/	convenience sampling	641	junior college students	35(5.46%)	606 (94.54%)	/
Yang et al. (77)	Anhui, China	/	Stratified random sampling	732	junior college students	40(5.5%)	692 (94.5%)	18.85 ± 1
Zheng et al. (101)	Guangdong, Dongguan, China	2022.5–6	Stratified random sampling	249	junior college students	33(13.25%)	216 (86.75%)	/
Fang et al. (102)	China	/	Stratified random sampling	1,335	junior college students	104(7.8%)	1,231 (92.2%)	21.51 ± 1
Wu (103)	Suzhou, Jiangsu, China	/	Random sampling	213	junior college students	29(13.62%)	184(86.38% )	20. 12 ± 1
Yan et al. (104)	Guizhou China	/	Cluster sampling	199	Nursing students	5(2.5%)	194 (97.5%)	/
KAOP 34–204 Alshehry et al. (85)	Saudi Arabia	2017.11–12	Total enumeration sampling	175	Bachelor of Science in Nursing (BSN)	81 (46.29%)	94 (53.71%)	19.84 ± 0
Ayoglu et al. (86)	Turkey	2011–2012 academic term	/	339	/	66 (19.47%)	273 (80.53%)	/
Chance et al. (87) (1)	United States	/	/	169	Pre-licensure nursing students	/	/	/
Chance et al. (87) (2)	Costa Rica	/	/	100	Pre-licensure nursing students	/	/	/
Cheng (88)	Hong Kong, China	2018.1–2	Convenience sample	139	Nursing students	34 (24.46%)	105 (75.54%)	
Darling et al. (43)	Turkey	/	Convenience sample	468	Undergraduate students	61 (13.03%)	407 (86.97%)	20.6 ± 1
Galzignato et al. (89)	Venice, Italy	2019–2020	/	383	Nursing degree	91 (23.76%)	292 (76.24%)	/
Ghimire et al. (44)	Kathmandu, Nepal	2017.11–2018.3	Random sampling	385	Bachelor of Science in Nursing (BSN) Bachelor of Nursing (BN)	0	385 (100.00%)	22.2 ± 3.2
Zhang et al. (28)	Heilongjiang , China	2020.12–2021.2	Purposive sampling	371	Junior college students	41 (11.05%)	330 (88.95%)	/
Zverev (91)	Malawian	/	Random sampling	151	Undergraduate students	28 (18.54%)	123 (81.46%)	/
Zhao et al. (84)	Hubei, China	/	/	258	Undergraduate students	/	/	/
Zhang et al. (28)	Zhengjiang, China	/	Convenience sample	230	Undergraduate students	33 (14.35%)	197 (85.65%)	/
Zheng et al. (94)	Hunan, Chin a	2016.5	Random-stratified cluster sampling	315	Undergraduate students, Junior college students	2(0.63%)	313 (99.36%)	20.87 ± 2
Pu et al. (105)	China	2013.9	Convenience sample	112	Undergraduate students	12 (10.71%)	100 (89.29%)	/
Ma et al. (106)	Gansu, Lanzhou, China	2018.1–3	Convenience sample	182	Undergraduate students, Junior college students,postgra duate students	17 (9.3%)	165 (90.7%)	/
Huang et al. (107)	Guangxi, China	2021.10–2022.5	Convenience sample	308	Undergraduate students, Junior college students	45 (14.6%)	263 (85.4%)	18–23
Ran et al. (108) and Erdemir et al. (109)	Chongqing, China	2017.11–2018.2	Convenience sample	393	vocational college students	16(4.07%)	377 (95.93%)	19.13 ± 1
	African, Turkey	/	/	455	Undergraduate students	165(36.3%)	290(63.7)	21.78 ± 2

For the KAOP scale with total scores ranging from 34 to 204, 17 studies encompassing 4,399 nursing students were included between 2011 and 2022. Nine studies (50%) (27, 83, 87) analyzed data from China, while two studies (16.7%) (47, 85) focused on Turkey. Additional studies incorporated data from the United States (86), Nepal (48), Malawi (90), Saudi Arabia, Costa Rica (86), and Italy (88). The sampling methods employed convenience sampling, random sampling, purposeful sampling, and random-stratified cluster sampling. Fifteen studies reported KAOP scores separately for male and female nursing students.

### 3.3 Risk of bias assessment

The assessment of study quality showed that three studies (47, 48, 88) (4.6%) were of high-quality, while 53 studies (8, 17, 18, 27–29, 31–33, 35–46, 51–54, 56–62, 64–67, 69–73, 75–83, 87, 89, 91) (81.5%) were of moderate quality, and eight studies (6, 15, 30, 55, 84–86, 90) were categorized as low quality. All studies explicitly stated their sources of information (item 1). In addition, more than 75% of the studies documented the population’s continuity (item 4), described assessments undertaken for quality assurance (item 6), and provided summaries of response rates and data completeness (item 10). However, only five studies explained the methods used to address missing data (item 9), and merely two studies discussed the potential influence of assessors’ subjective factors on the findings (item 5).

Furthermore, 74% of the studies did not clarify patient exclusion criteria (item 7), while approximately 50% failed to report any items (items 2, 3, and 8).

### 3.4 Meta-analysis results

**Figure 2:**
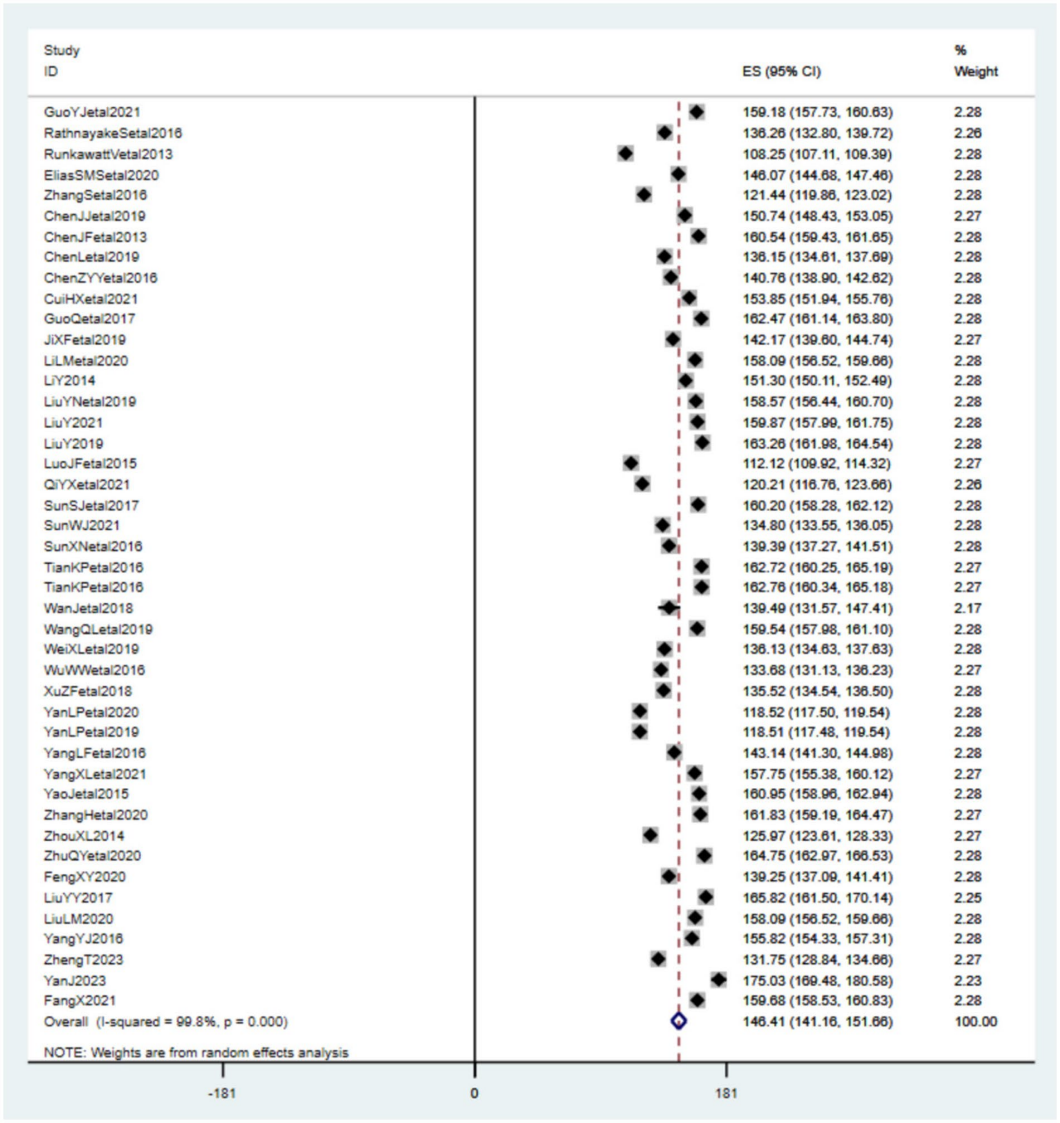
Forest plot of the attitudes of nursing students toward the older adults using the KAOP 34–238 scale.

For the 47 studies reporting KAOP scale total scores ranging from 34 to 238, the random effects model analysis yielded a mean KAOP score of 146.41 (95% CI: 141.16–151.66, *I*^2^ = 99.8%, *p* < 0.001). A forest plot is presented in Figure 2.

**Figure 3:**
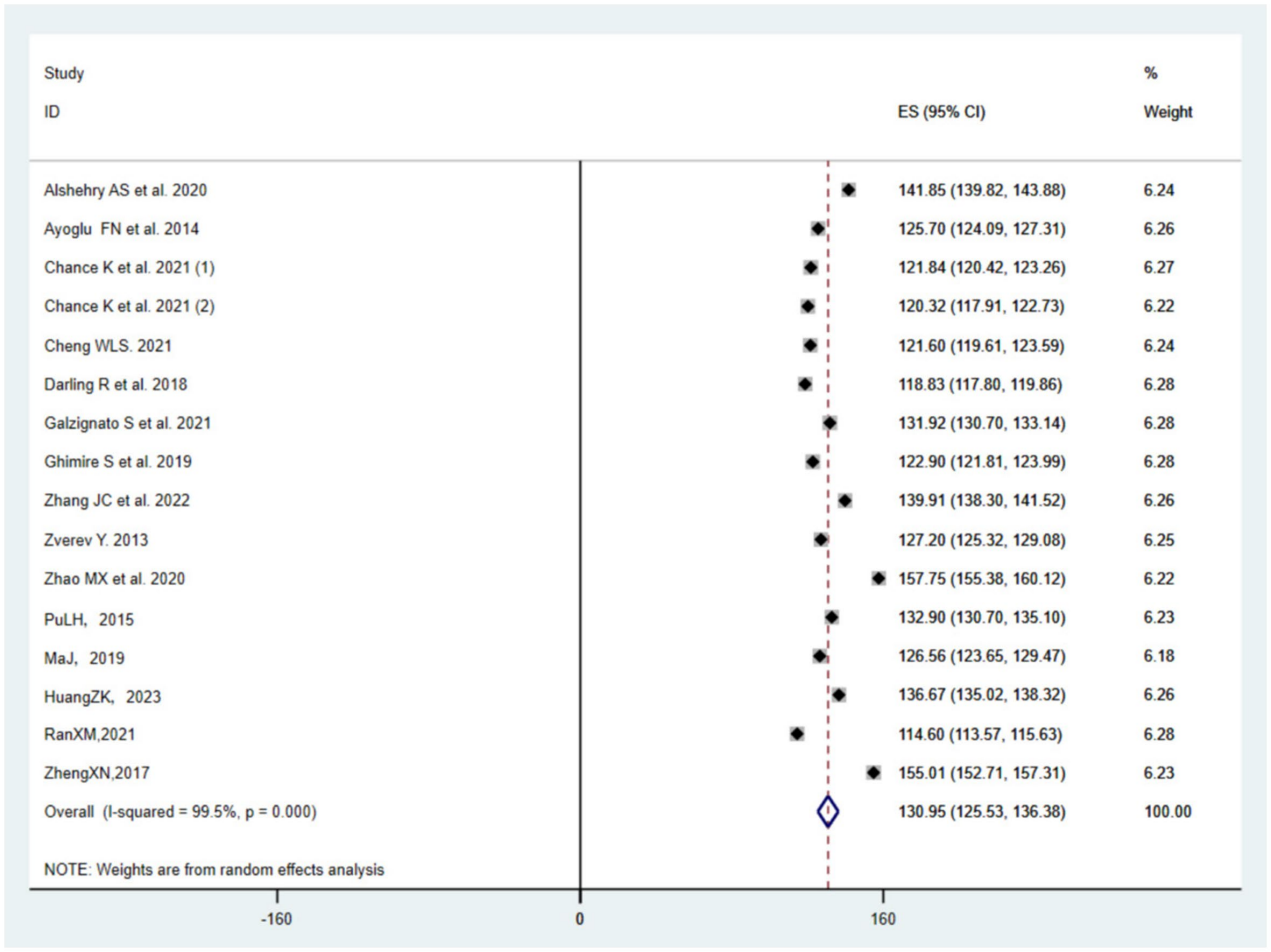
Forest plot of the attitudes of nursing students toward the older adults using the KAOP 34–204 scale.

For the 17 studies with KAOP scale total scores ranging from 34 to 204, the random effects model analysis showed that the mean KAOP scores of nursing students were 130.95 (95% CI: 125.53–136.39, *I*^2^ = 99.5%, *p* = 0.000). Figure 3 presents the corresponding forest plot.

#### 3.4.1 Subgroup analysis

Subgroup analyses for the nine predictor variables identified as significant in the meta-regression revealed the following pooled estimates of nursing students’ attitudes toward geriatric care. Formal between subgroup tests (meta-regression) showed that the following factors were statistically significant moderators: gender (*p* = 0.03), living with older adults (*p* = 0.01), caring for older adults (*p* = 0.002), geriatric training course (*p* = 0.04), nursing as first choice (*p* = 0.02), and academic year (*p* = 0.01). Student origin (*p* = 0.68) and only-child status (*p* = 0.54) were not significant.as measured by the total KAOP scale scores of 34–238; Gender: Male (144.00, 95% CI = 138.81–149.19) vs. Female (149.41, 95% CI = 143.49–155.33); Student Origin: Urban (151.41, 95% CI = 144.32–158.50) vs. Rural (150.53, 95% CI = 143.49–157.57); Only-child status: Yes (151.32, 95% CI = 144.16–158.48) vs. No (150.39, 95% CI = 143.37–157.41); Experience of living with older adults: Yes (152.77, 95% CI = 147.11–158.43) vs. No (149.06, 95% CI = 143.45–154.68); Experience of caring for older adults: Yes (156.75, 95% CI = 151.67–161.84) vs. No (151.72, 95% CI = 145.77–157.68); Nursing as first career choice: Yes(151.42, 95% CI = 138.00–164.84) vs. No (145.74, 95% CI = 133.42–158.05); completion of a geriatric training course: Yes (139.36, 95% CI = 122.97–155.75) vs. No (136.63, 95% CI = 124.922–148.33); Year level: First-year (144.57, 95% CI = 125.72–163.41), Second-year (151.70, 95% CI = 145.70–157.69), Third- and Fourth-year (152.82, 95% CI = 148.08–157.56); Country of study: China (147.60, 95% CI = 142.53–152.66) vs. Other countries (130.18, 95% CI = 102.055–158.31).

From the total KAOP scale scores of 34–204, the three predictor variables provided the following composite estimates of nursing students’ attitudes toward involvement in caring for older adults: Gender: Male (132.27, 95% CI = 126.84–137.6) vs. Female (131.81, 95% CI = 125.75–137.87); Having experience in caring for older adults: Yes (138.32, 95% CI = 127.85–148.79) vs. No (134.72, 95% CI = 118.26–151.18); Year Level: First-year (133.83, 95% CI = 127.07–140.59), Second-year (135.20, 95% CI = 125.50–144.91), and third- or fourth-year (137.52, 95% CI = 131.23–143.81).

Further details are provided in Table 2.

#### 3.4.2 Sensitivity analysis

In the sensitivity analysis, we found that no individual estimate significantly influenced the overall effect. For the 47 studies with a KAOP scale total scores of 34–238, the sensitivity analysis showed that the KAOP scores of nursing students ranged from 141.16–151.67. Similarly, for the 17 studies with KAOP scale total scores of 34–204, the KAOP scores ranged from 126.68–131.08.

#### 3.4.3 Publication bias

**Figure 4:**
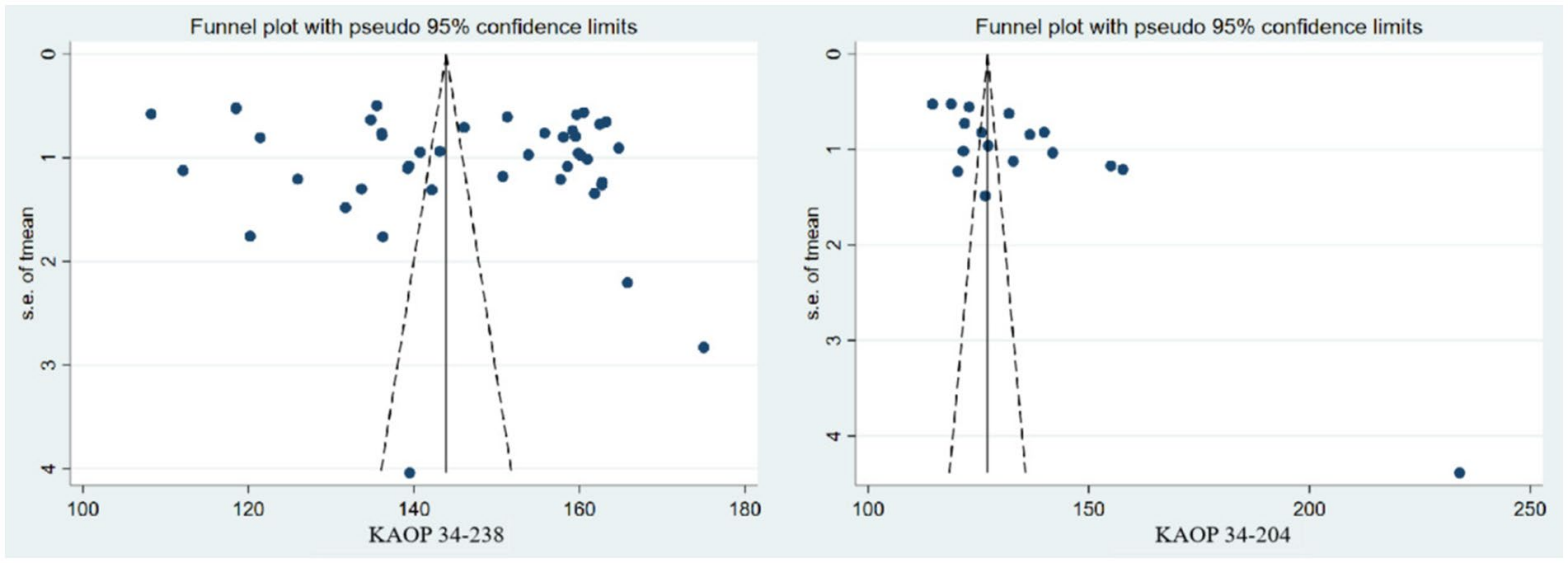
Funnel plot of publication bias.

The results of Egger’s test indicated no publication bias for studies reporting KAOP scores of 34–238 among nursing students (*p* = 0.165, *p* > 0.05). However, Egger’s test was statistically significant (*p* = 0.011, *p* < 0.05) for KAOP scores of 34–204, suggesting that small-study effects or publication bias may have influenced the observed results. The funnel plot is shown in Figure 4.

## 4 Discussion

Sixty-four cross-sectional studies involving 24,332 nursing students were included in this study, of which 82.8% were from China, and 87.5% were assessed as medium to high quality. The primary factors affecting study quality included the failure to explain the impact of the assessors’ subjective factors on their conclusions, the lack of transparency in methods used to handle missing data, and the omission of explanations for patient exclusions. The meta-analysis revealed that the total KAOP 34–238 score of nursing students regarding attitudes toward older adults was 146.41 (95% CI: 141.16–151.66, *I*^2^ = 99.8%, *p* < 0.001), while the total KAOP 34–204 score was 130.95 (95% CI: 125.53–136.39, *I*^2^ = 99.5%, *p* < 0.001).

The subgroup analysis revealed that female nursing students exhibited more positive attitudes toward caring for older adults than their male counterparts, a finding supported by multiple studies (34, 92). This difference may stem from gender-role socialization, wherein caregiving is traditionally associated with femininity, potentially leading male nursing students to perceive geriatric nursing as less aligned with their professional identity. This may contribute to gender disparities in attitudes toward older adults.

Nursing students with experience caring for or living with older individuals exhibited significantly more positive attitudes toward older adults. This finding is not surprising, as direct interaction with older individuals allows students to develop a deeper understanding of their living conditions and needs. Such experiences may foster trust in older adults, build positive attitudes, dispel misconceptions and stereotypes, and help students develop a more empathetic and objective perspective. However, as the included studies are cross-sectional, reverse causality cannot be ruled out. Therefore, fostering meaningful contact between nursing students and older individuals seems to play a crucial role in promoting positive attitudes. For example, nursing schools could establish long-term collaborations with geriatric care institutions, providing students with stable clinical placements. Regular internships, volunteer opportunities, and participation in home care services could enable students to gain first-hand insights into the real challenges of geriatric care. These initiatives would increase student interaction opportunities, promoting a more informed and compassionate approach to geriatric nursing.

Education shapes nursing students’ attitudes toward geriatric care (93). Studies indicate that students who have taken geriatric nursing courses exhibit significantly more positive attitudes than those who have not. This may be because exposure to geriatric education enhances students’ understanding of aging-related issues. Strengthening geriatric nursing education is a crucial strategy for improving students’ knowledge and attitudes toward aging. Emphasizing geriatric nursing content in curricula is essential for preparing future healthcare professionals to meet the needs of an aging population. A study by Duan et al. found that introducing a geriatric care course fostered positive attitudes toward older adults among nursing students (94). Similarly, Husebo et al. reported that positive experiences in geriatric nursing learning education increase students’ confidence in pursuing careers in geriatric care (42). Some countries, such as the United States, have incorporated geriatric nursing competencies into their registered nurse licensure examinations, which may encourage nursing students to enhance their knowledge and skills in geriatric care (95).

Nursing students who selected nursing as their first-choice career were more actively involved in geriatric care than those who did not. This difference may be attributed to the stronger professional identity of students who were committed to nursing from the outset of their education. Career choices and personal values significantly influence professional identity. Therefore, fostering a sense of professional belonging among nursing students is essential. This can be achieved by nurturing their professional identity, strengthening their professional thoughts and attitudes regarding nursing, and instilling a sense of dedication and responsibility toward gerontological care. Encouraging these attitudes may increase students’ willingness to pursue careers in geriatric nursing after graduation (96).

The subgroup analysis of nursing students’ academic levels revealed that fourth-year students exhibited the most positive attitudes toward older adults, while first-year students scored the lowest. Many studies have shown that a higher level of knowledge about aging correlates with more positive attitudes toward geriatric care. Generally, fourth-year students have completed geriatric nursing courses and participated in numerous voluntary and practical activities related to geriatric care, contributing to their greater knowledge of geriatric issues. Additionally, senior students face imminent employment decisions, which may lead to a more mature and pragmatic career perspective under the pressure of job placement.

This study has some limitations. First, although we conducted a comprehensive search of Chinese and English databases, 82% of the included data were from China, which may introduce geographical bias. Future research should investigate regional differences by incorporating data from a broader range of geographical, economic, and demographic backgrounds. Second, this study is a systematic review of cross-sectional studies, which inherently carry a greater degree of heterogeneity and potential bias. Therefore, future studies should employ prospective cohort designs with larger, more representative populations.

## 5 Conclusion

The findings indicated that nursing students generally hold positive attitudes toward caring for older adults. Key influencing factors included gender, experience of living with or caring for older individuals, selecting nursing as a first-choice career, and academic level. However, further high-quality studies with larger, more representative samples are needed to validate these findings.

## Data Availability

The datasets presented in this study can be found in online repositories. The names of the repository/repositories and accession number(s) can be found in the article/Supplementary material.
