# Supplementary material for: Attitudes of nursing students toward older adults: a systematic review and meta-analysis
Source: Front Public Health. 2026 Jun 4;14:1813734. doi: 10.3389/fpubh.2026.1813734 (PMC13275347; doi:10.3389/fpubh.2026.1813734)
Supplement: Supplementary file 1 [file Supplementary_file_1.docx]

Supplementary Appendix 1 ： Search Strategy

| Databases [Platform] | Results |
| --- | --- |
| PubMed (*July 06, 2024*) | 493 |
| Web of Science (*July 06, 2024*) | 735 |
| Embase (*July 24, 2022*) | 490 |
| Cochrane Library (*July 06, 2024*) | 249 |
| CNKI (*July 06, 2024*) | 280 |
| WANFANG (*July 06, 2024*) | 3644 |
| CBM(*July 06, 2024*) | 207 |
| TOTAL | 6098 |
| After Removing Duplications | 5685 |

| Databases | Set | Search terms | Items found |
| --- | --- | --- | --- |
| PubMed | 1 | "Students, Nursing"[Mesh] | 31944 |
|  | 2 | "undergraduate nurs*"[Title/Abstract] OR "nursing undergraduat*"[Title/Abstract] OR "nursing student*"[Title/Abstract] OR "student nurs*"[Title/Abstract] OR "bsn student*"[Title/Abstract] OR "bachelor of nursing student*"[Title/Abstract] OR "baccalaureate nursing student*"[Title/Abstract] OR "student nurse"[Title/Abstract] OR "bachelor"[Title/Abstract] OR "pre-licensure baccalaureate"[Title/Abstract] | 32483 |
|  | 3 | **1 OR 2** | 46538 |
|  | 4 | "Geriatric Nursing"[Mesh] | 13916 |
|  | 5 | "geriatric care"[Title/Abstract] OR "geriatric nurs*"[Title/Abstract] OR "aged care"[Title/Abstract] OR "gerontology nurs*"[Title/Abstract] OR "old people"[Title/Abstract] OR "elderly"[Title/Abstract] OR "old age"[Title/Abstract] OR "older adults"[Title/Abstract] OR "older population"[Title/Abstract] OR "elder care"[Title/Abstract] | 460206 |
|  | 6 | **4 OR 5** | 469109 |
|  | 7 | "intent*"[Title/Abstract] OR "attitude"[Title/Abstract] OR "perception*"[Title/Abstract] OR "knowledge"[Title/Abstract] OR "belief*"[Title/Abstract] OR "opinion*"[Title/Abstract] OR "preference*"[Title/Abstract] | 1841941 |
|  | **8** | **3 AND 6 AND 7** | **493** |
| Web of Science | 1 | TS= ("undergraduate nurs*" or "nursing undergraduat*" or "nursing student*" or "student nurs*" or "bsn student*" or "bachelor of nursing student*" or "baccalaureate nursing student*" or "student nurse" or "bachelor" or "pre-licensure baccalaureate") | 46650 |
|  | 2 | TS= ("geriatric care" or "geriatric nurs*" or "aged care" or "gerontology nurs*" or "old people" or "elderly" or "old age" or "older adults" or "older population" or "elder care") | 759249 |
|  | 3 | TS= ( "intent*" or "attitude" or "perception*" or "knowledge" or "belief*" or "opinion*" or "preference*") | 5106075 |
|  | **4** | **1 AND 2 AND 3** | **735** |
| Cochrane Library | 1 | MeSH descriptor: [Students, Nursing] explode all trees | 851 |
|  | 2 | (undergraduate nurs*):ti,ab,kw OR (nursing undergraduat*):ti,ab,kw OR (nursing student*):ti,ab,kw OR (student nurs*):ti,ab,kw OR (bsn student*):ti,ab,kw OR (bachelor of nursing student*):ti,ab,kw OR (baccalaureate nursing student*):ti,ab,kw OR (student nurse):ti,ab,kw OR (bachelor):ti,ab,kw OR (pre-licensure baccalaureate):ti,ab,kw | 4614 |
|  | 3 | **1 OR 2** | 4614 |
|  | 4 | MeSH descriptor: [Geriatric Nursing] explode all trees | 209 |
|  | 5 | (geriatric care):ti,ab,kw OR (geriatric nurs*):ti,ab,kw OR (aged care):ti,ab,kw OR (gerontology nurs*):ti,ab,kw OR (old people):ti,ab,kw OR (elderly):ti,ab,kw OR (old age):ti,ab,kw OR (older adults):ti,ab,kw OR (older population):ti,ab,kw OR (elder care):ti,ab,kw | 229130 |
|  | 6 | **4 OR 5** | 229130 |
|  | 7 | (intent*):ti,ab,kw OR (attitude):ti,ab,kw OR (perception*):ti,ab,kw OR (knowledge):ti,ab,kw OR (belief*):ti,ab,kw OR (opinion*):ti,ab,kw OR (preference*):ti,ab,kw | 192190 |
|  | **8** | **3 AND 6 AND 7** | **249** |
| Embase | 1 | 'nursing student'/exp | 35147 |
|  | 2 | 'undergraduate nurs*':ab,ti OR 'nursing undergraduat*':ab,ti OR 'nursing student':ab,ti OR 'student nurs*':ab,ti OR 'bsn student*':ab,ti OR 'bachelor of nursing student*':ab,ti OR 'baccalaureate nursing student*':ab,ti OR 'student nurse':ab,ti OR bachelor:ab,ti OR 'pre-licensure baccalaureate':ab,ti | 19519 |
|  | 3 | **1 OR 2** | 44959 |
|  | 4 | 'geriatric nursing'/exp | 13233 |
|  | 5 | 'geriatric care':ab,ti OR 'geriatric nurs*':ab,ti OR 'aged care':ab,ti OR 'gerontology nurs*':ab,ti OR 'old people':ab,ti OR elderly:ab,ti OR 'old age':ab,ti OR 'older adults':ab,ti OR 'older population':ab,ti OR 'elder care':ab,ti | 614977 |
|  | 6 | **4 OR 5** | 623488 |
|  | 7 | intent*:ab,ti OR attitude:ab,ti OR perception*:ab,ti OR knowledge:ab,ti OR belief*:ab,ti OR opinion*:ab,ti OR preference*:ab,ti | 2285862 |
|  | **8** | **3 AND 6 AND 7** | **490** |
| CNKI |  | 主题=(护生 + 护士生 + 护理学生 + 护理专业学生) AND 主题=(老人 + 老年人 + 老年护理) AND主题=(意愿 + 态度 + 意向 + 倾向) | **288** |
| WANFANG |  | 主题:(护生 or 护士生 or 护理学生 or 护理专业学生) AND 主题:(老人 or 老年人 or 老年护理) AND主题:(意愿 or 态度 or 意向 or 倾向) | **3644** |
| CBM | 1 | "学生, 护理"[不加权:扩展] | 23916 |
|  | 2 | "护生"[常用字段:智能] OR "护士生"[常用字段:智能] OR "护理学生"[常用字段:智能] OR "护理专业学生"[常用字段:智能] | 37624 |
|  | 3 | **1 OR 2** | 37624 |
|  | 4 | "老人"[常用字段:智能] OR "老年人"[常用字段:智能] OR "老年护理"[常用字段:智能] | 190022 |
|  | 5 | "态度"[不加权:扩展] | 53886 |
|  | 6 | "意愿"[常用字段:智能] OR "态度"[常用字段:智能] OR "意向"[常用字段:智能] OR "倾向"[常用字段:智能] | 169010 |
|  | 7 | **5 OR 6** | 169010 |
|  | **8** | **3 AND 4 AND 7** | **207** |
